# Membrane Separation Process in Wastewater and Water Purification

**DOI:** 10.3390/membranes12030259

**Published:** 2022-02-24

**Authors:** Alexandre Giacobbo, Andréa Moura Bernardes

**Affiliations:** Post-Graduation Program in Mining, Metallurgical and Materials Engineering (PPGE3M), Federal University of Rio Grande do Sul (UFRGS), Av. Bento Gonçalves n. 9500, Agronomia, Porto Alegre 91509-900, RS, Brazil

The current scenario of increasing water scarcity and degradation of water bodies has led to the development of processes and technologies that provide more suitable treatment for both water and wastewater. Among the possible technologies used, membrane separation processes (MSPs) have gained prominence compared to other technologies thanks to their inherent characteristics such as low energy requirement, mild operating conditions, no additives, separation efficiency, and easy scaling up, as well as for providing greater security to the treatment. In addition, in the case of wastewater treatment, depending on the technology adopted, it is still possible to obtain high-quality water, which can be reused in different activities, as well as the recovery of resources from the wastewater.

In this regard, the Special Issue entitled “*Membrane Separation Process in Wastewater and Water Purification*” aims to assess in-depth recent developments and advances in different aspects related to sustainable and environmentally friendly membrane technology. Various subjects are discussed, all associated with optimal operation parameters for water/wastewater treatment. The removal of organic compounds from water are especially discussed in two works, including cementitious membrane fabrication and its application with catalytic ozonation for the removal of small molecule organic pollutants, and a case-study addressing the effect of pH on the transport of a pharmaceutical compound through polyamide nanofiltration membranes. Two other works addresses membrane fouling, one on coagulation–membrane rotation in drinking water treatment, and another about ultrafiltration for direct digestate purification. One work deals with porous membrane wall hydrocyclone for oily water treatment, and another one presents an ecological and carbon footprint assessment in a reverse osmosis plant. Therefore, this Special Issue features the contribution of six research articles, and a brief descriptive summary of the scientific contributions follows.

In the first study, from cementitious powders and amorphous silica (C-10 µm), Sun et al. [1] developed a cementitious microfiltration membrane (CM) with a catalytic ozone oxidation function for the removal of organic pollutants. By increasing the silica–cement ratio (s/c) the authors observed that the membranes showed an increase in pure water flux (PWF) and a decrease in mechanical strength. An s/c equal to 0.5 was chosen as the optimal ratio for manufacturing the CM, as the membrane presented the ideal combination of mechanical strength and PWF, in addition to the highest porosity. The authors observed that the CMs were rich in surface hydroxyl groups and had an alkaline buffering effect, so that within minutes, the pH of the solution rose to values in the range from 10 to 11.5 and remained constant. For this reason, in subsequent experiments, CMs were shown to have the ability to catalyze the ozonation process in the degradation of organic pollutants such as nitrobenzene, benzophenone-4, *p*-chlorophenol, *p*-chloronitrobenzene, and *p*-chlorobenzoic acid, a fact that gives support for practical applications of CMs in the degradation of organic contaminants.

Considering that fouling is still the main drawback of membrane technology in water treatment, Yu et al. [2] investigated the impact of combining coagulation and ultrafiltration (UF) membrane rotation on membrane fouling control and removal of organic matter (macro- and micro-pollutants) in drinking water treatment. They verified an effective reduction in membrane fouling, especially that caused by macro-organic pollutants, a fact that was attributed to shear and vortex stresses caused by membrane rotation. Furthermore, membrane fouling can be further reduced by the incorporation of a coagulation step, which provided the aggregation of the organic substances in flocs and their elimination by membrane retention. Thus, the study showed that the integration of coagulation and membrane rotation is beneficial for mitigating fouling and prolonging the membranes’ lifetime in drinking water treatment.

Additionally, concerning the importance of optimizing operating conditions for a better performance of membrane technology, Yue et al. [3] assessed the effects of membrane molecular weight cut-off (MWCO), transmembrane pressure (TMP), temperature, and cross-flow velocity (CFV) on permeate flux, permeate quality, and fouling characteristics during ultrafiltration of anaerobic digestate from swine manure. They reported that all four membranes evaluated (MWCO of 5, 10, 20, and 50 kDa) showed chemical oxygen demand (COD) removals greater than 78% and were able to effectively remove tetracycline residues—antibiotics commonly used in livestock. The authors also found a relationship between the increase in permeate flux with the increase in CFV, temperature, and membrane MWCO, and that the higher the membrane MWCO, the higher the limit flux. In addition, they reported that C, O and S were the main elements responsible for membrane fouling, while *Pseudomonadales*, *Xanthomonadales* and *Burkholderiales* bacteria were the predominant ones. On the other hand, cleaning procedures with NaOH and NaClO were more efficient in removing the fouling layer from the membrane than those with citric acid and tap water.

Nunes et al. [4] investigated the applicability of a hydrocyclone with a porous membrane wall for the separation of a water–oil mixture. The hydrocyclone studied has two tangential inlets (on top) and two concentric outlets (permeate and concentrate) at the bottom of the equipment. The authors used the computational fluid dynamics technique in a Eulerian–Eulerian approach to solving the mass and linear momentum conservation equations and the turbulence model. They evaluated the effects of the membrane rejection coefficient (*R*) and concentration polarization layer thickness on the permeate flux, hydrodynamic behavior of the fluids inside the hydrocyclone, and equipment performance. Based on the results obtained, the authors concluded that there was no significant variation in the hydrodynamic behavior of the fluids inside the hydrocyclone when it was operated with rejection coefficients in the range of 0.96 ≤ *R* < 1.00, thus demonstrating the high potential of this equipment for separating oil–water mixtures.

The solution–diffusion model may be used to describe the solute–membrane interactions in nanofiltration (NF), in such a way that a parameter *B* is used to quantify the affinity of a given solute for the membrane. In that case, *B* $=\frac{J_{P}\left(1-f_{A}^{\prime }\right)}{f_{A}^{\prime }}$, where $J_{P}$ and $f_{A}^{\prime }\;$ are the permeate flux and the intrinsic rejection coefficient for the solute *A*, respectively. In this context, Soares et al. [5] studied the influence of pH on the removal of a pharmaceutical compound, Atenolol (ATN), by nanofiltration, as well as its effect on the affinity of this pharmaceutical compound to the membrane by determining the parameter *B*. Two commercial NF membranes (NF90 and NF270), four transmembrane pressures (6, 8, 10 and 12 bar), and three different pH solutions (2.5, 7.0 and 10.5) were tested. It was observed that the solution pH affected the performance of the membranes, promoting repulsion or attraction between the ATN and the membranes. At pH 2.5, the lowest values for parameter *B* were obtained, which is associated with the low-affinity ATN–membranes, since both membranes and ATN presented positive charges, causing electrostatic repulsion, and consequently, greater rejections of ATN. In this sense, the authors emphasized the importance of determining parameter *B* together with the optimization of the hydrodynamic conditions and other operational parameters, in order to better understand the mechanisms of NF separation and above all to contribute to optimizing the process and obtaining the best cost–benefit.

Lastly, Leon et al. [6] studied the ecological and carbon footprints in a reverse osmosis sea water desalination plant located on the island of Tenerife, specifically in the municipality of Granadilla de Abona (Canary Islands, Spain). The study was carried out taking into account the technical characteristics of the desalination plant, the power generation, and the carbon dioxide fixation capacity per hectare in the Canary Islands. Among the conclusions presented, one can highlight that: (i) the ecological footprint of Granadilla de Abona is 0.065–0.087 Gha/inhabitant/year; (ii) the energy consumption decreases as the temperature of the feed water increases and when its salinity decreases; (iii) the use of renewable energies reduces emissions and carbon footprint; (iv) Canary Islands desalination carbon footprint is around 439,402 tCO_2_/year and the ecological footprint is 219,701 ha/year.

In fact, thanks to the increasing developments in terms of membrane materials, membrane modules, optimization of operating and hydrodynamic conditions, adoption of adequate pretreatments, as well as integration with other technologies, the use of membrane separation processes has been boosted in a variety of applications, especially in those related to water and wastewater treatment. In the frame of this scenario, the present Special Issue contributes six research articles that provide an overview of the different strategies available to achieve a better performance of membrane separation processes in water and wastewater treatment.

## Data Availability

Not applicable.
